# Rapid Detection of the Activity of *Lacticaseibacillus* Casei Zhang by Flow Cytometry

**DOI:** 10.3390/foods12061208

**Published:** 2023-03-12

**Authors:** Xuebo Ma, Lina Wang, Lixia Dai, Lai-Yu Kwok, Qiuhua Bao

**Affiliations:** 1 Key Laboratory of Dairy Biotechnology and Engineering, Ministry of Education, Inner Mongolia Agricultural University, Hohhot 010018, China; 2 Key Laboratory of Dairy Products Processing, Ministry of Agriculture and Rural Affairs, Inner Mongolia Agricultural University, Hohhot 010018, China; 3 Inner Mongolia Key Laboratory of Dairy Biotechnology and Engineering, Inner Mongolia Agricultural University, Hohhot 010018, China

**Keywords:** *Lacticaseibacillus casei* Zhang, activity detection, flow cytometry, fluorescent dyes, plate count, CFDA, propidium iodide

## Abstract

Food processing, e.g., freeze-drying, exerts strong pressure on bacteria in the food matrix, decreasing their viability/activity and even forcing them to become viable but unculturable (VBNC), which are often underestimated by traditional plate count. The strict standards of bacterial viability in probiotic products require accurate cell viability/activity enumeration. We developed a staining (5(6)-carboxyfluorescein diacetate succinimide ester, propidium iodide)-based flow cytometry rapid method for detecting the viability/activity of *Lacticaseibacillus* (*Lb*.) *casei* Zhang, a widely used probiotic in the dairy industry in China. We optimized the procedural and instrumental parameters for generating results comparable to that of standard plate counts. This method was also applied to freeze-dried *Lb. casei* Zhang, yielding 7.7 × 10^11^ CFU/g, which was non-significantly higher than the results obtained by plate count (6.4 × 10^11^ CFU/g), possibly due to the detection of VBNC cells in the freeze-dried powder. We anticipated that this method can be used for detecting lactic acid bacteria in other probiotic food/beverages.

## 1. Introduction

Lactic acid bacteria are a group of bacteria that use fermentable carbohydrates to produce large amounts of lactic acid [1], which include genera such as *Lactobacillus*, *Lactococcus*, *Pediococcus*, *Enterococcus*, and *Streptococcus* [2]. *Lacticaseibacillus* (*Lb.*) *casei* Zhang is a widely studied probiotic strain isolated from koumiss in Inner Mongolia, China. It is resistant to acid, bile salts, and oxidation stress and has been shown to improve the gut microbiota in humans [3]. It is also widely used in fermented milk, solid beverages, medicine, and aquaculture [4,5,6].

Currently, traditional plate colony counting is the gold standard for quality assurance of the viability of probiotics in products [7]. The measurement relies on the ability of bacterial cells to grow in a liquid medium and on solid agar plates for absorbance detection or colony counting, respectively [8]. However, in food processing and production, probiotic bacteria encounter various environmental stresses, including exposure to extreme temperatures, acids, and unfavorable osmotic pressure [9]. These damaging external factors cause temporary or complete damage to the cells and/or cellular function, consequently changing the cell morphology and physiology. Bacterial cells in finished fermented products often appear as a mixture of healthy and vulnerable cell populations of altered states. For example, some of them enter a sublethal state, i.e., viable but non-culturable (VBNC), which are cells that exhibit and survive with a low level of activity for an extended period [10,11,12]. The current gold standard methods of evaluating probiotic viability rely mainly on enumerating bacterial growth, which fails to detect VBNC bacteria due to their vulnerability and difficulty in recovery for growth [13], leading to the underestimation of the probiotic viability in fermented products. Other disadvantages of the traditional approach include cumbersome experimental preparation and time consumption. Therefore, there is a need for developing rapid methods for accurate assessment of the bacterial activity of lactic acid bacteria that undergo different food processing conditions.

Different methods have been developed to overcome the limitations of traditional microbiological techniques in the past decades [14]. For example, flow cytometry (FCM) can detect and distinguish between live, damaged, and dead bacterial cells within a mixed population when used in combination with specific fluorescent dyes that stain cells based on physiological state and metabolic activities. Dyes used in FCM for live/dead cell distinction are generally divided into two categories: membrane permeable and non-permeable. Permeable dyes can penetrate intact membranes and bind to cellular nucleic acids [15]. For example, SYBR Green binds to DNA, and SYTO 9 binds to DNA and RNA, both fluorescing green, while 5(6)-carboxyfluorescein diacetate succinimide ester (CFDA) binds to intracellular proteins and labels intact cells. Double staining with 6-carboxy-fluorescein diacetate (CFDA) and propidium iodide (PI) can be used to check bacterial enzyme activity. CFDA is a kind of intracellular esterase substrate, which is widely used as an indicator of bacterial esterase activity [16]. CFDA is usually a non-fluorescent, non-polar derivative, which easily penetrates the cell membrane. Intracellular CFDA is hydrolyzed by a functional cytoplasmic enzyme, which, if present and active, leads to the conversion of esterase into 6-carboxy-fluorescein and release. Additionally, 6-Carboxyfluorescein is negatively charged at physiological pH, so it accumulates in cells with an intact plasma membrane. Therefore, the cells stained by CFDA have esterase activity, and the complete cell membrane [17] 6-carboxy-fluorescein shows bright green fluorescence under blue light irradiation, has polarity, and remains in the cells. PI has polarity and can only penetrate the inactive cell membrane. PI is an impermeable dye used to detect dead cells because it can only enter cells through damaged cell membranes. After the PI in the cell is combined with double-stranded DNA [18], it emits bright red fluorescence under blue light excitation. Therefore, after double staining with CFDA and PI, after blue light irradiation, esterase active bacterial cells only display a green 6-carboxy-fluorescein signal, while inactivated cells only display red PI fluorescence. Therefore, bacterial cells with and without esterase activity can be accurately distinguished. The differential permeating abilities, binding specificities, and excitation and emission wavelengths of these dyes make them informative tools in FCM applications.

The US Pharmacopoeia has approved FCM as a tool for cell-based product development and quality control [19]. FCM is an effective method for bacterial cell count and viability determination, which is widely used in medicine, microbiology, on-site environmental detection, and other fields [20,21,22]. For example, in medical research, Kalina T and other [23] people reported the effective use of FCM in primary immunodeficiency, immunophenotype, diagnosis, and functional research; in microbial research, Leonard L and other [24] people discussed the potential opportunities provided by FCM in detecting the impact of antibiotics on microbial subpopulations; and in environmental testing, some scholars used FCM to evaluate the effect of disinfectants in water on fungal spores and to study their inactivation mechanism [25]. In recent years, the detection of microorganisms in food by flow cytometry has become more and more extensive. In one study, rod-shaped curd spores of *Bacillus coagulans* MTCC 5856 were added to a commercial orange juice, and the number of spores was estimated by the plate colony counting method and FCM after 24 h. The FCM method showed a more accurate result, which was much closer to the actual spore number compared with the estimation by the plate colony counting method (approximately one logarithm lower), confirming the high accuracy of the FCM method in detecting dormant cells in commercial products [26].

The function of probiotic products is largely based on the viability of the probiotics in the final products; thus, the detection of viable cells in these products must be accurate. Indeed, there are strict legal requirements for cell viability in probiotic products. Provided the labor intensiveness and insensitivity of recovering less active cells, such as VBNC, traditional cultivation methods might not be ideal for the rapid detection of probiotic bacteria in commercial food products. This study aimed to establish an FCM-based method for detecting lactic acid bacteria after food processing steps. Freeze drying is a common method used in the food process and preparation of dairy starters. Thus, herein, we established an FCM-based rapid method to detect live monoculture and freeze-dried *Lb. casei* Zhang. We envisage that the developed FCM method can have a broad application for detecting and quantifying live lactic acid bacteria in other food products.

## 2. Materials and Methods

### 2.1. Bacterial Strain and Culture Conditions

The probiotic strain *Lb. casei* Zhang was preserved in and provided by the Lactic Acid Bacteria Collection Center, the Ministry of Education Key Laboratory of Dairy Products and Biotechnology, Inner Mongolia Agricultural University. The strain has been deposited in the China General Microbiological Culture Collection Center (Deposit No. 1697). The pure strain was stored in 10% skimmed milk plus 0.1% sodium glutamate at −80 °C.

### 2.2. Strain Activation

To activate the frozen stock, *Lb. casei* Zhang was inoculated in liquid MRS medium and cultured at 37 °C in a constant-temperature incubator (HWS28, Heng yi Science Instrument Co., Ltd., Shanghai, China). After reaching the logarithmic growth phase, the initial culture was subcultured (2%) twice before further experiments were carried out. All cultures were assessed for strain purity via observation of cell morphology by optical microscopy (DM4000B Leica, Wetzlar, Germany).

### 2.3. Preparation of Lb. casei Zhang Culture

Standard plate colony counting method was used to determine the number of bacteria in the *Lb. casei* Zhang culture (found to be 10^9^ colony-forming units [CFU]/mL), which was then serially diluted to 10^2^, 10^3^, 10^4^, 10^5^, 10^6^, 10^7^, 10^8^, and 10^9^ CFU/mL, respectively. Then, 3 mL of each bacterial dilution was washed and resuspended in the same volume of phosphate-buffered saline. Then, each tube was split into three 1 mL aliquots. The first aliquot was processed for FCM using a CytoFLEX benchtop flow cytometer (Beckman Coulter, Inc., Indianapolis, IN, USA); cells were incubated at 37 °C with CFDA (10 μL of 5 mmol/L) for 15 min followed by incubation with PI (10 μL of 1.5 mmol/L) for another 15 min in the dark. The second aliquot was used as an FCM negative control (no addition of fluorescent dyes). The third aliquot was used for plate colony counting. The plate colony counting and FCM counting methods were compared with respect to the detection range and bacterial density.

### 2.4. Freeze-Drying of Lb. casei Zhang

The same *Lb. casei* Zhang bacterial culture for FCM analysis was pelleted by centrifugation, and the supernatant was removed. The bacterial pellet was transferred to a 50 mL centrifuge tube to be frozen at −80 °C for 16 h followed by freeze-drying (FD-1A-50, Beijing Boyekang Experimental Instrument Co., Ltd., Beijing, China) for 20 h (20 Pa, −45 °C).

### 2.5. Optimization of FCM Conditions

#### 2.5.1. Optimization of Dye Addition and Dyeing Time

The dye concentration and dyeing time are critical for proper FCM detection of bacterial cells; insufficiency of either parameter results in poor fluorescence signals and erroneous results. The experimental design was based on the estimation by the SPSS software (25 SPSS Inc., Chicago, IL, USA) to ensure the coverage of an ample range of dye concentrations and dyeing times (Table 1 and Table 2). The effects of varying these variables on the detection results were analyzed.

#### 2.5.2. Optimization of Sample Loading Speed and Time Combination

Cell counting by FCM is based either on a standard microsphere counting method or a volume method, and this study employed the volume method. Basically, the actual number or concentration of target cells in a sample was determined using the optical characteristics of the cells and the sample volume [27]. In FCM, the sample loading volume directly affects the measurement accuracy, and the sample loading speed and time are key factors that affect the loading sample volume. Thus, it would be necessary to optimize the loading speed and time combination.

Stained samples were detected by FCM using different loading speeds and times. According to the CytoFLEX manual, a sample volume of 10 μL or greater would produce the optimal result. A fixed loading volume of 10 μL was tested with a combination of different detection speeds ranging from 10 to 60 μL/min (stepwise increase of 10 μL until an upper limit of 60 μL/min to avoid data distortion) for data acquisition.

#### 2.5.3. Optimization of Threshold

The threshold in FCM defines an obstacle or a limit, and only signals greater than this threshold were recorded [28]. The threshold of each channel (forward scatter, FSC; side scatter, SSC) can be set based on individual samples to reasonably acquire the most encompassing data. For example, data are recorded if the detected signal from the cell sample is greater than the set threshold in the FSC channel. If the threshold is set too high or low, part of the fluorescence signal is lost, and the results are skewed. Careful threshold adjustment can eliminate unnecessary signal noise and ensure that most of the relevant data are collected. Here, the FSC and SSC channel thresholds were set to 10, 100, 1000, 10,000, and 50,000, respectively. The most appropriate threshold values were selected in the optimized method.

#### 2.5.4. Gain Optimization

The FCM signal value can be fine-tuned by adjusting the instrument’s gain; increasing the gain will enhance the signal, and reducing the gain will weaken the signal. When the resolution of a channel is very low, the gain can be increased to separate negative from weak positive signals; if the positive fluorescence signal is too strong, the gain value can be reduced. These adjustments ensure that all positive cells are displayed in the output chart and make data processing convenient. Therefore, we optimized the gain of the FSC and SSC channels, as well as the FITC and PE fluorescence channels.

### 2.6. Detection of the Viability/Activity of Freeze-Dried Lb. casei Zhang

Lyophilized *Lb. casei* Zhang bacterial powder, prepared as described above, was enumerated using the plate colony counting method and found to contain ~10^11^ CFU/g. The bacterial powder (100 mg) was added to 9.9 mL phosphate-buffered saline and serially diluted to 10^6^ CFU/mL. This diluted sample was used for both plate colony counting and FCM.

### 2.7. Statistics

The results from three experimental replicates were analyzed for statistical significance by analysis of variance (ANOVA) using IBM SPSS Statistics 25 (SPSS Inc., Chicago, IL, USA). Spearman’s correlation analysis was performed with IBM SPSS Statistics 25 to assess the correlation between results generated by plate counting and FCM detection. The extreme value analysis of R indicated that dyeing time, PI addition amount, and CFDA addition amount affected the detection of viable bacteria.

## 3. Results and Discussion

### 3.1. Determination of Lb. casei Zhang Culture Purity by Optical Microscopy

**Figure 1:**
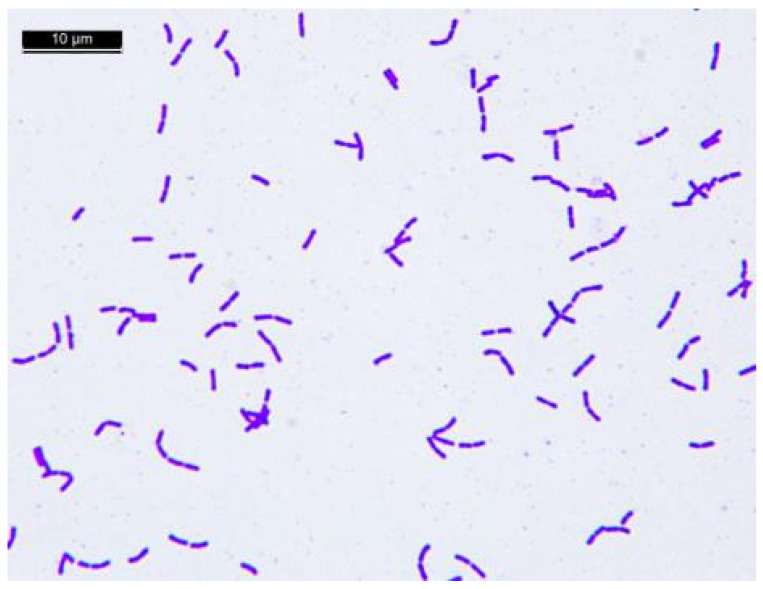
Gram stain cell morphology of *Lacticaseibacillus casei* Zhang (scale = 10 μm).

Under a light microscope, Gram-stained *Lb. casei* Zhang cells were found to be short-to-medium rods, as expected. As seen in Figure 1, there were no miscellaneous bacteria in the field of vision, indicating that the *Lb. casei* Zhang culture was pure.

### 3.2. Detection of Different Cell Densities of Lb. casei Zhang by FCM and Plate Count

**Figure 2:**
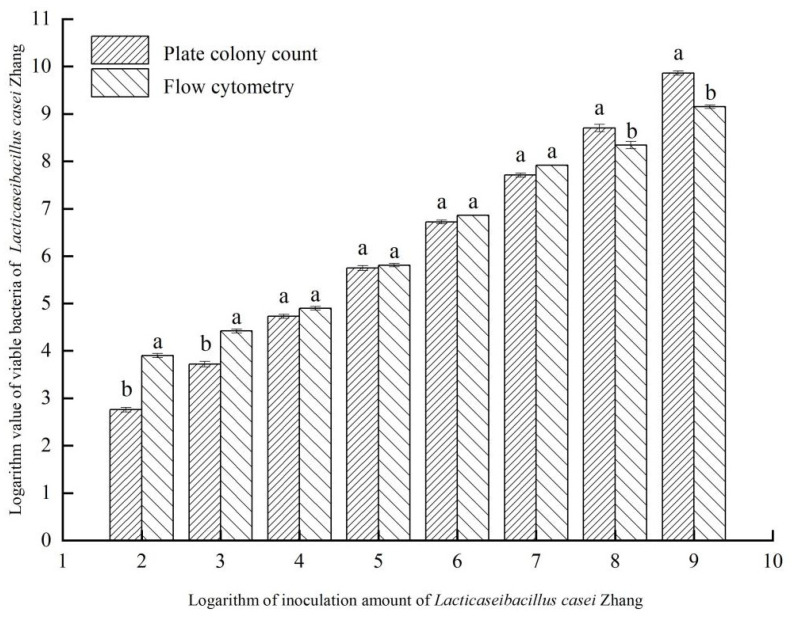
Comparison between flow cytometry and plate colony counting in enumerating *Lacticaseibacillus* (*Lb.*) casei Zhang. Different letters written above the bars indicate significant differences (*p* < 0.05, T-student) between two detection methods at the same cell density. Bars represent means ± SD with no statistical differences between both methods and use letters a and b when there are statistical differences.

Serially diluted cultures of *Lb. casei* Zhang that contained 10^2^, 10^3^, 10^4^, 10^5^, 10^6^, 10^7^, 10^8^, or 10^9^ CFU/mL were detected by plate colony counting and FCM concurrently. The results of plate colony counting and FCM detection showed a strong positive correlation in the cell density ranging from 10^4^ to 10^7^ CFU/mL (*r* = 0.984, *p* < 0.01; Figure 2), indicating consistent and accurate measurement by FCM detection. However, relatively large deviations were observed between the two detection methods at a low (10^2^ to 10^3^ CFU/mL) or high (10^8^ to 10^9^ CFU/mL) cell density range. Therefore, the optimal range for cell density determination was 10^4^ to 10^7^ CFU/mL, which was similar to the ideal bacterial concentration range determined in previous studies [29]. A bacterial density of 10^6^ CFU/mL was chosen for subsequent experiments in this study due to its highest consistency between the detection methods among other dilutions.

### 3.3. Determination of the Dye Concentration and Dyeing Time

Using an orthogonal experimental design, the optimal combination of fluorescent FCM dye concentration and incubation time was determined empirically (Table 3). The influence of these factors on the detection of viable bacteria was as follows: dyeing time t1 > dyeing time t2 > PI addition > CFDA addition. The optimal combination producing the best detection results was a CFDA amount of 10 μL, dyeing time (t1) of 15 min, PI amount of 5 μL, and dyeing time (t2) of 15 min. Under these conditions, the viable count of *Lb. casei* Zhang reached 1.897 active fluorescent units (AFU)/μL, equivalent to 1.897 × 10^9^ CFU/mL.

### 3.4. Optimal Combination of Sample Loading Speed and Time

**Figure 3:**
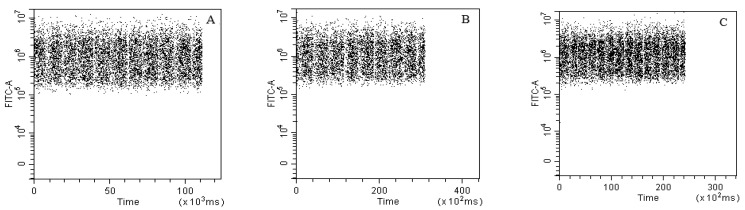
Flow cytometer flow rate fluorescence signal dot plots. (**A**) Low speed; (**B**) medium speed; and (**C**) high speed.

To ensure the accuracy of FCM data, the flow rate of the sample must first be optimized. A poor flow rate during detection would result in signal interruption or instability. Therefore, signal detection at low, medium, and high flow rates was investigated (Figure 3). The stability of the fluorescence signal was relatively good at all tested speeds, as there was no obvious disconnection or other impeding phenomena indicating signal instability during detection.

**Figure 4:**
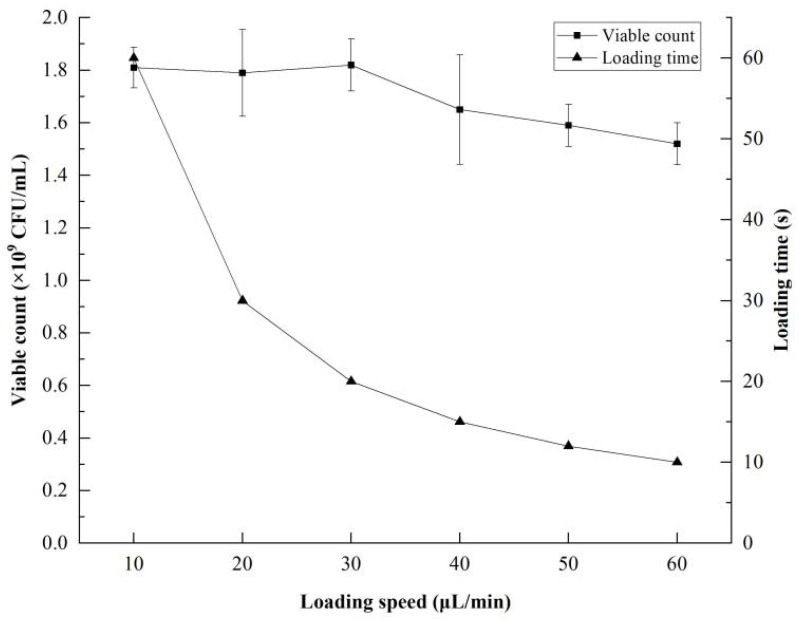
Influence of sample loading speed on the result of viable count detection. Different letters represent significant differences in viable count between different loading speeds. Error bars represent SD.

Next, the optimal combination of sample loading speed (the time needed to load 10 μL) and detection time was determined using serially diluted viable bacteria. The sample loading speeds tested were 10, 20, 30, 40, 50, and 60 μL/min, respectively, and the loading times tested were 10, 20, 30, 40, 50, and 60 s. The maximum FCM detection of viable bacteria occurred at a sample loading speed of 30 μL/min, corresponding to a loading time of 20 s; these parameter settings also showed the best agreement between the FCM results and the known CFU/mL values of the bacterial samples (Figure 4).

### 3.5. Threshold Calibration

**Figure 5:**
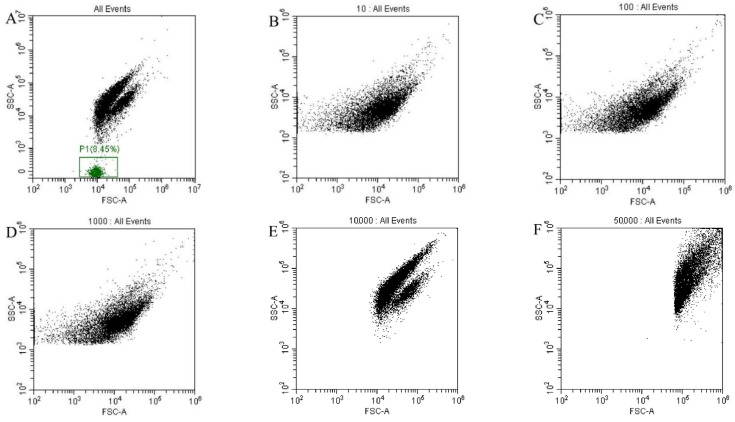
Application of different thresholds for FSC channel. (**A**) When a signal channel (FSC or SSC) was used for data collection. (**B**–**F**) FSC set to (**B**) 10; (**C**) 100; (**D**) 1000; (**E**) 10,000; and (**F**) 50,000.

Unwanted signal noise can be eliminated by adjusting the instrument’s threshold settings, ensuring that most of the desired signals are properly collected. When a single channel was used for data collection, more background noise was observed in the P1 area (Figure 5A). Thus, both FSC and SSC were used for data collection. First, different thresholds were set in the FSC channel. Signals obtained from the FSC channel set to 10, 100, 1000, 10,000, and 50,000 showed that a threshold setting of less than 10,000 could not separate signals from the sample from the impurity (noise). A threshold setting of 10,000 showed good clustering of the sample signal, while a threshold setting of 50,000 resulted in the loss of the sample signal (Figure 5B–F). The FSC channel threshold setting was therefore set to 10,000.

**Figure 6:**
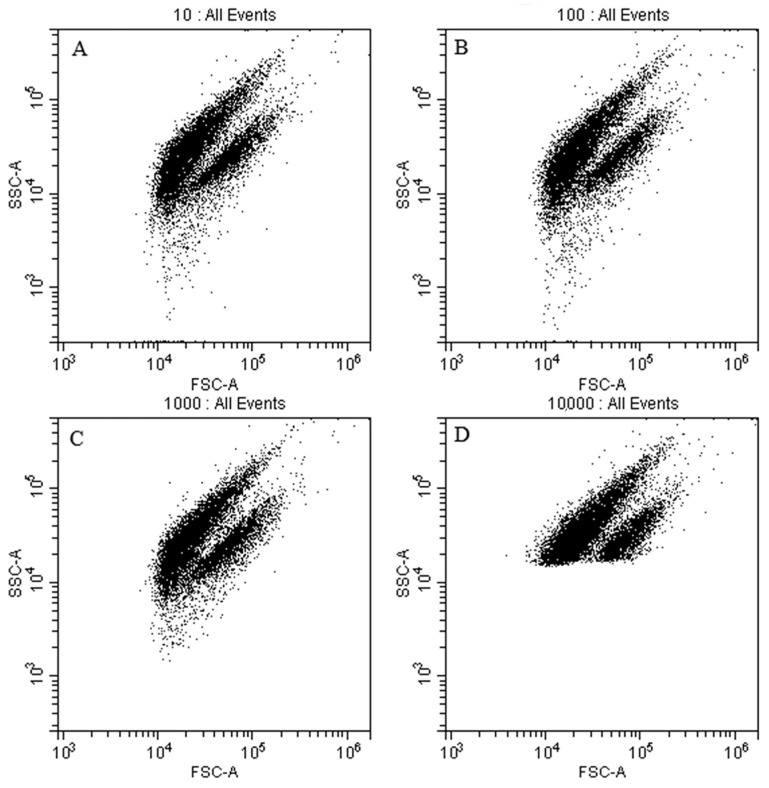
Application of different thresholds of SSC channel. SSC set to (**A**) 10; (**B**) 100; (**C**) 1000; and (**D**) 10,000.

The SSC channel threshold was then optimized by setting its threshold to the range of 10 to 10,000, respectively (Figure 6A–D). The setting to a value of 10,000 resulted in a significant loss of some sample fluorescence signals, but lower threshold settings increased the noise signals. The SSC channel threshold was kept at 1000 (Figure 6C).

### 3.6. Gain Calibration

**Figure 7:**
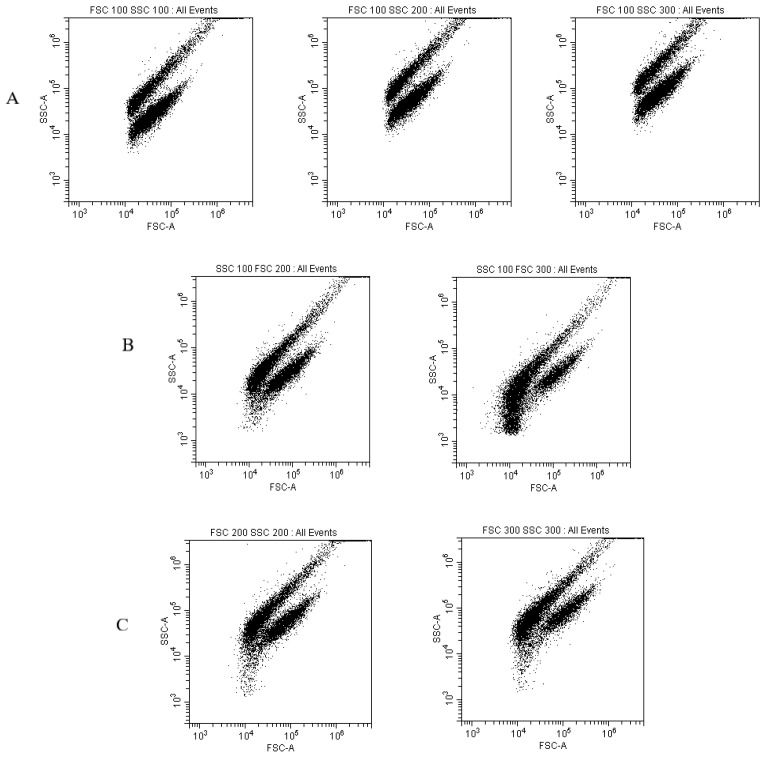
Different combinations of gain settings of FSC and SSC channels. (**A**) FSC, 100; SSC 100–300; (**B**) FSC, 200; SSC, 200–300; and (**C**) FSC, 200–300, SSC, 200–300.

The gain settings of the FSC and SSC channels were also calibrated. The fluorescence signal was partially lost or not fully displayed when an FSC channel gain setting of 100 and SSC channel gain setting of 100, 200, or 300 were used. When three SSC settings were used, there was almost no difference in signals (Figure 7A). Conversely, when an SSC channel gain setting of 100 was used, the two signals approached more with an increased FSC gain level of 300 versus 100 and 200 due to parameter changes in the coil gate (Figure 7B). When the gain settings of both the FSC and SSC channels were increased to 200 and 300 concurrently, the two signals were also approaching (Figure 7C); however, too high gain settings can easily damage the instrument. Therefore, FSC and SSC channel gain settings of 200 and 100 were chosen, respectively.

**Figure 8:**
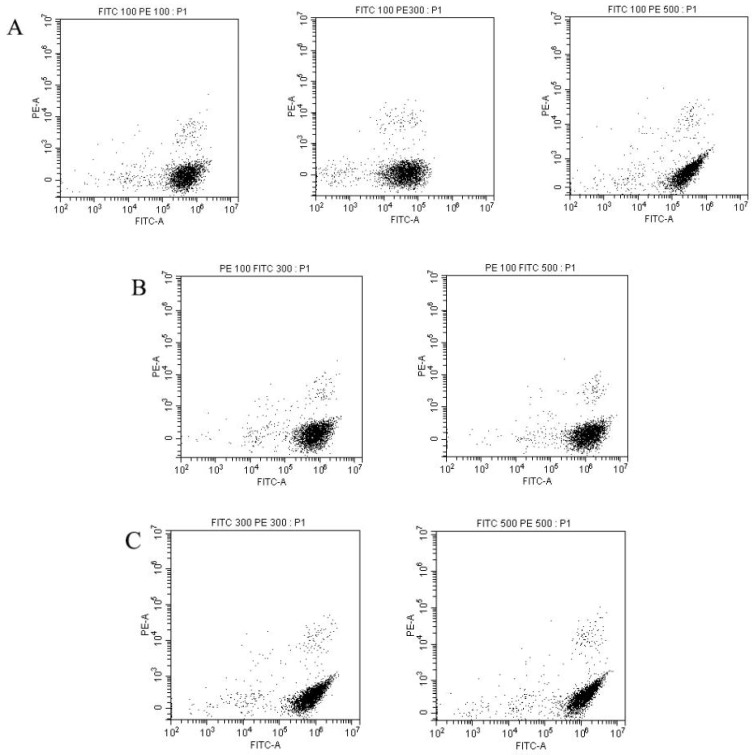
Different combinations of gain settings of FITC and PE channels. (**A**) FITC, 100; PE, 100, 300, and 500; (**B**) PE 100; FITC, 300, 500; and (**C**) FITC, 300, 500; PE 300, 500.

Next, the gain of the FITC and PE channels was calibrated. First, the gain of the FITC channel was set to 100, while the gain of the PE channel was set to 100, 300, or 500, respectively (Figure 8A); the best result was observed when the PE channel gain was set to 300 for easy distinction between positive and negative signals (Figure 8A). Secondly, the gain of the PE channel was set to 100, while the gain of the FITC channel was set to 300 or 500, respectively, and there were few variations between the two settings (Figure 8B). Similarly, no significant differences were observed in the dispersals of detected signals on the dot plots when both the FITC and PE channel gains were set to 300 or 500 simultaneously (Figure 8C). Therefore, the FITC and PE gains were set to 100 and 300, respectively.

### 3.7. Detection of Lb. casei Zhang Bacterial Powder

Based on the results of procedure optimization, the most suitable cell density of *Lb. casei* Zhang in FCM detection was 10^6^ CFU/mL. The optimized staining procedures were CFDA (10 μL) incubated with the sample at 37 °C for 15 min followed by a 15 min incubation with PI (5 μL) in the dark. The optimal instrumental settings were a sample loading speed and time of 30 μL/min and 20 s, respectively; FSC and SSC channel thresholds of 10,000 and 1000, respectively; FSC and SSC channel gains of 200 and 100, respectively; and FITC and PE channel gains of 100 and 300, respectively. Freeze-dried bacterial powder of *Lb. casei* Zhang was resuspended at 10^6^ CFU/mL for quantification by FCM. An aliquot of the bacterial powder was stained as described above; meanwhile, the negative control without staining was processed in parallel. All sample measurements were performed in triplicate using the optimized instrument parameters. The number of viable *Lb. casei* Zhang cells detected was 7.7 ± 0.17 (×10^11^ CFU/g) by FCM and 6.4 ± 0.45 (×10^11^ CFU/g) by the concurrent plate colony counting method.

**Figure 9:**
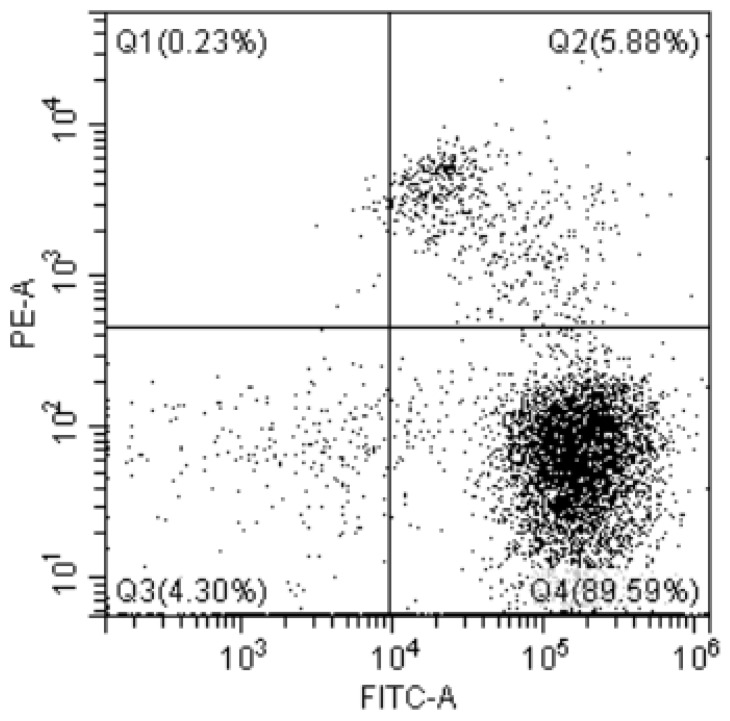
Distribution of dead and live bacteria detected by *Lb. casei* Zhang bacterial powder. Q1: dead bacteria; Q2: dormant and injured cells. cells (VBNC cells); Q3: impurity; and Q4: viable cells with intact membrane.

The two detection methods found a similar range of *Lb. casei* Zhang viable cells (within the same order of magnitude) with a slightly higher value found in the FCM detection compared with the plate colony counting. Previously, FCM consistently detected lactic acid bacteria activity in yogurt and yak milk [30]. The subtle discrepancy between the results of FCM and plate colony count can be explained by several reasons. First, the detection principles and units of expression differ. Traditional plate colony counting is expressed in CFU/g, which represents the number of living bacteria that could form colonies on solid media. Some bacterial cells could be closely distributed on the agar plates and fused together, appearing as one single colony, which leads to the underestimation of cell number. The bacterial powder was tested using FCM to determine the distribution of live bacteria, as showed in Figure 9. The FCM method detects AFU, which would also count the VBNC due to their viability and basal activity, though some of these cells might not be recovered by routine plate count culture. The mechanically damaged cells may be VBNC cells, which need further verification. The slightly higher number of live bacteria detected by FCM may be related to this.

## 4. Conclusions

In this study, we optimized the procedural and instrumental conditions for detecting *Lb. casei* Zhang, widely used probiotics in dairy products, by FCM in a combination of cellular staining. We showed that this method could also detect freeze-dried bacterial cells, as it bases on the staining of cell activity, achieving a slightly higher sensitivity than traditional plate counting. Our study established a rapid method for quantification of lactic acid bacteria that might be at stress or in the VBNC state due to food processing, such as freeze-drying. We anticipated that the developed FCM method could be applied to the detection and quantification of live lactic acid bacteria in other food products.

## Figures and Tables

**Table 1:** Orthogonal experimental factor level table of dye addition and dyeing time.

Level	Amount of CFDA Added (μL)	Dyeing Time t1 (s)	Amount of PI Added (μL)	Dyeing Time t2 (s)
1	5	5	5	5
2	10	10	10	10
3	15	15	15	15

Remarks: CFDA = 5(6)-carboxyfluorescein diacetate succinimide ester; PI = propidium iodide.

**Table 2:** Orthogonal experimental design of dye addition amount and dyeing time.

Serial Number	Amount of CFDA Added (μL)	Dyeing Time t1 (s)	Amount of PI Added (μL)	Dyeing Time t2 (s)
1	15	15	10	5
2	10	10	15	5
3	15	5	15	15
4	10	15	5	15
5	5	10	10	15
6	15	10	5	10
7	10	5	10	10
8	5	5	5	5
9	5	15	15	10

Remarks: CFDA = 5(6)-carboxyfluorescein diacetate succinimide ester; PI = propidium iodide.

**Table 3:** Orthogonal test for identifying the optimal combination of dye concentration and dyeing time.

Experiment	Parameters				Viable Count (AFU/μL)
	A CFDA Amount (μL)	B Dyeing Time t1 (s)	C PI Amount (μL)	D Dyeing Time t2 (s)	
1	5	5	5	5	1661
2	5	10	10	15	1762
3	5	15	15	10	1802
4	10	15	5	15	1897
5	10	5	10	10	1782
6	10	10	15	5	1739
7	15	5	15	15	1628
8	15	15	10	5	1786
9	15	10	5	10	1824
K1	1742	1691	1794	1729	
K2	1806	1775	1777	1803	
K3	1746	1829	1723	1763	
R	64	138	71	74	
Factor ranking	B > D > C > A				
Optimal combination	A2B3C1D2				

## Data Availability

All related data and methods are presented in this paper. Additional inquiries should be addressed to the corresponding author.
